# Anti-Mullerian as predictor of reproductive outcome in infertile women: follow up

**DOI:** 10.5935/1518-0557.20140012

**Published:** 2014

**Authors:** Juliano B. Scheffer, Bruno B. Scheffer, Rafaela F. de Carvalho, Laura T. Vellez, Fabio Florencio, Michael Grynberg

**Affiliations:** 1 IBRRA - Instituto Brasileiro de Reprodução Assistida, Belo Horizonte, MG, Brazil; 2 Department of Reproductive Medicine, Hôpital Jean Verdier (AP-HP), University Paris XIII, and INSERM, U782, Clamart - France

**Keywords:** Ovarian stimulation, pituitary suppression, IVF, ICSI, pregnancy

## Abstract

**Objective:**

The aim of the present study was to investigate and to compare the relations of anti-Mullerian with the prognostic parameters and the outcome of assisted reproductive treatment.

**Methods:**

Prospective longitudinal study. A total of one hundred and twelve infertile women. Inclusion criteria: i) both ovaries present, ii) no current or past diseases affecting ovaries or gonadotropin or sex steroid secretion, clearance, or excretion, iii) no current hormone therapy, iv) adequate visualization of ovaries at transvaginal ultrasound scans, and v) total number of small antral follicles (3-12 mm in diameter) between 1 and 32 follicles. On cycle day 3, woman underwent blood sampling for serum FSH and AMH measurement and a transvaginal ovarian ultrasound scan for follicle measurement. Ongoing pregnancy was evaluated as biochemical pregnancy and observation of gestational sac(s).

**Results:**

Mean age of 36.13 ± 4.65 years old, BMI 21.59 ± 2.78 kg/m2, and length of infertility of 2.88 ± 2.36 years. Their ovaries had an average of 13.74 ± 6.0 antral follicles and AMH was 2.49 ± 1.98 ng / mL. A significant relationship of AMH with age (r =-0.37 *P* <.01), with FSH (r =-0.22, *P* <.01), with AFC (r = 0.74, *P* <.00001), with smoking (*P* <.009), with SOP (*P* <.00001), with the total dose of the drug during stimulation ovarian (r =-0.36, *P* <.0004), with abortion (*P* <.05) and with the ongoing pregnancy (*P* <.05).

**Conclusion:**

AMH is a marker of quantitative and qualitative aspects of the ovarian reserve.

## INTRODUCTION

Anti-Müllerian hormone (AMH), also called Müllerian Inhibiting substance, is a dimeric glycoprotein, belonging to the transforming growth factor-b (TGF-b) superfamily, such as activins and inhibins, and is produced exclusively in the gonads, as shown more than two decades ago in animals (Vigier *et al*., 1984) and later in humans (Rey *et al.*, 2003; Di Clemente *et al.*, 1992). In women, AMH is synthetized by the granulosa cells (GC) surrounding preantral and small antral follicles (Weenen *et al.*, 2004; Durlinger *et al*., 2002). Despite the use of ultrasensitive assays, AMH is barely detectable in the serum at birth. It reaches higher levels after puberty (Guibourdenche *et al.*, 2003; Rajpert-De Meyts *et al*., 1999) and then declines with advancing female age, until becoming undetectable again at the time of the menopause (La Marca *et al.*, 2005). Although its physiological roles and the mechanisms involved in the regulation of AMH still remain poorly established, recent studies have pointed out this hormone as an attractive marker for assessing of ovarian activity.

Basal AMH, determined before stimulation (usually cycle day 3), was found to be a better measure for the assessment of a decreased ovarian reserve and the ovarian response to controlled ovarian hyperstimulation (COH) with gonadotropins (Anckaert *et al.*, 2012; Nardo *et al.*, 2009) when compared to the classic parameters such as an increase in follicle stimulating hormone (FSH), the decrease of inhibin B, or the antral follicle count (Fanchin *et al.*, 2005; Tremellen *et al.*, 2005; Hazout, 2006). It has also been shown that AMH was inversely correlated, in addition to age, to basal FSH values (Piltonen *et al.*, 2005). AMH has been claimed to possess at least the same level of accuracy as the antral follicle count (AFC) for the prediction of poor and excessive (Broer *et al.*, 2011) response. In addition, a high serum concentration of AMH before the start of COH has been shown to be associated with increased risk of developing ovarian hyperstimulation syndrome (OHSS) (Nardo *et al.*, 2009). As with other ovarian reserve tests, AMH has not proven to be a good predictor of embryo quality or pregnancy in COS cycles, suggesting that AMH is a marker of quantitative rather than qualitative aspects of the ovarian reserve (Anckaert *et al.*, 2012; Broer *et al.*, 2011).

The association between the ovarian response and the basal AMH production was confirmed by other studies, showing an increased yield of mature oocytes after controlled stimulation in women presenting high serum AMH levels (Fleming *et al*., 2006; Smeenk *et al*., 2007; Ebner *et a*l., 2006; Ficicioglu *et al*., 2006).

The aim of the present study was to investigate and to compare the relations of AMH with the prognostic parameters and the outcome of assisted reproductive technology (ART).

## MATERIAL AND METHODS

### Subjects

One hundred and twelve infertile women, aged 22-50 years, undergoing routine exploration during an unstimulated cycle that preceded ART at our center were studied prospectively, from February 2009 to December 2012. All patients met the following inclusion criteria: i) both ovaries present, ii) no current or past diseases affecting ovaries or gonadotropin or sex steroid secretion, clearance, or excretion, iii) no current hormone therapy, iv) adequate visualization of ovaries at transvaginal ultrasound scans, and v) total number of small antral follicles (3-12 mm in diameter) between 1 and 32 follicles, including both ovaries. All patients signed an informed consent form for this analysis.

### Protocol

The patients received leuprolide acetate (Lupron, Abbott, France), the GnRH-agonist was initiated at a dose of 2,0 mg per day during the midluteal phase with approximately a 5-day overlap with the OCP (Diane 35, Schering, Brasil). Pituitary down-regulation was monitored and patients with adequate pituitary desensitization started their recombinant FSH regime (Gonal-F; Merck-Serono Pharmaceuticals, Italy) and the dose of GnRH-agonist was reduced to 1.0 mg per day. FSH was started with dosages between 150 and 300 IU daily for 4 days with or without human menopausal gonadotropin (hMG) (Menopur; Ferring Pharmaceuticals, Germany). There after, the dose of FSH was individually adjusted according to the estradiol (E2) response and vaginal ultrasound findings.

When two follicles reached ≥16 to 18 mm, 250 mg, recombinant human Chorionic Gonadotropin (Ovidrel, Merck-Serono Pharmaceuticals, Italy) was administered and oocyte retrieval occurred 35 to 36 hours later.

Intracytoplasmatic sperm Injection (ICSI) was routinely performed in all the fertilization procedures. Fertilization was evident when two pronuclei were observed. Embryos were cultured until the day of transfer (day 3) in IVF Global® media (Life Global, Canada) supplemented with 10 % synthetic serum substitute (SSS) and graded by Veeck’s (Veeck, 1999) and Hsu (Hsu *et al.*, 1999) criteria before transfer.

The ET number was determined by following the Federal Council of Medicine - Brazil (FCM) guidelines. Luteal phase was supported with micronized P4, 600 mg/ day, administered continuously by vaginal route, starting on the evening of ET.

Ongoing pregnancy (OP) was evaluated as biochemical pregnancy (BQ) and subsequent observation of gestational sac(s). Abortion defined as a clinically recognized pregnancy loss before 20 weeks’ gestation.

### Hormonal Measurements and Ultrasound Scans

On cycle day 3 of the cycle preceding COH, each woman underwent blood sampling by venipuncture for serum AMH, and FSH measurement and a transvaginal ovarian ultrasound scan for follicle measurement.

Serum levels of AMH and FSH were determined using an automated multianalysis system with chemiluminescence detection (ACS-180; Bayer Diagnostics, Puteaux, France). Serum AMH levels were determined using a second generation enzyme-linked immunosorbent assay. Intra- and inter- assay coefficients of variation (CV) were < 6 and <10% respectively, lower detection limit at 0,13 ng/mL and linearity up to 21 ng/mL for AMH. For FSH, functional sensitivity was 0.1 mIU/mL, and intra-assay and interassay CV were 3% and 5%, respectively. Ultrasound scans were performed using a 3.7-9.3MHz multifrequency transvaginal probe (RIC5-9H; General Electric Medical Systems, Paris, France) by a single operator who was blinded to the results of hormone assays.

The objective of ultrasound examination was to evaluate the number and size of small antral follicles. Follicles measuring 3-12 mm in mean diameter (mean of two orthogonal diameters) in both ovaries were considered.

To optimize the reliability of ovarian follicular assessment, the ultrasound scanner was equipped with a tissue harmonic imaging system (Thomas & Rubin, 1998), which allowed improved image resolution and adequate recognition of follicular borders. Intra-analysis CV for follicular and ovarian measurements were <5%, and their lower limit of detection was 0.1 mm. In an effort to evaluate the bulk of granulosa cells in both ovaries, we calculated the mean follicle diameter (cumulative follicle diameter divided by the number of follicles measuring 3-12 mm in diameter in both ovaries) and the largest follicle diameter.

### Ethical approval

Written informed consent was obtained from all participants before inclusion. The study was approved by IBRRA Ethical Committee.

### Statistical Analysis

Descriptive parameters and patient characteristics were reported as mean SD or median (range) depending on the distribution.

The Student’s t-test was performed for continuous variables Wilcoxon and Pearson’s Test were used where appropriate for categorical variables. *P* <.05 was considered statistically significant.

## RESULTS

Overall, at the time of the investigation, patients had a mean age of 36.13 ± 4.65 years old, BMI 21.59 ± 2.78 kg/m2, and length of infertility of 2.88 ± 2.36 years. 79% had regular cycles and 3.6% were smokers. On cycle day 3, serum AMH level was 2.49 ± 1.98 ng/mL. At baseline, women had 13.74 ± 6.0 antral follicles.

**Figure 1:**
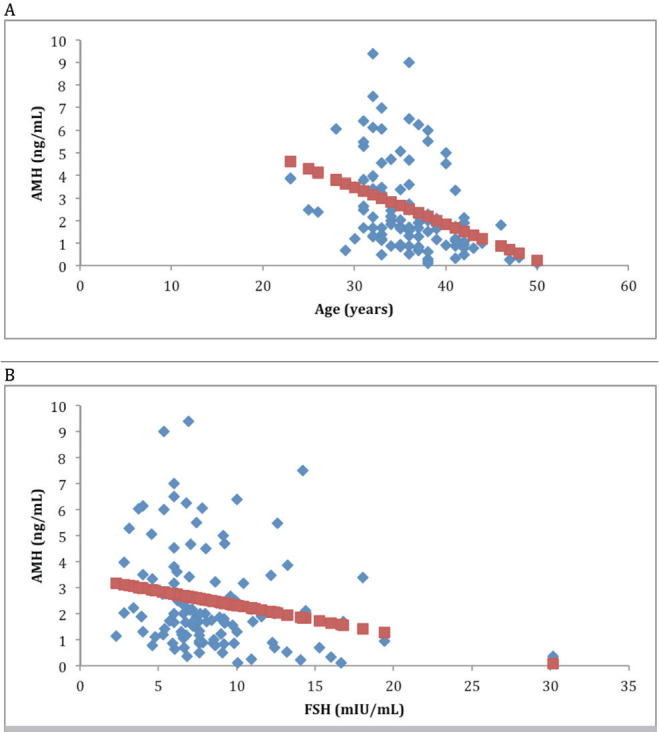
(A) Correlation between Anti-Mullerian Hormone (AMH) and age (B) Correlation between Anti-Mullerian (AMH) and day-3 Follicle Stimulating Hormone level (FSH).

**Figure 2:**
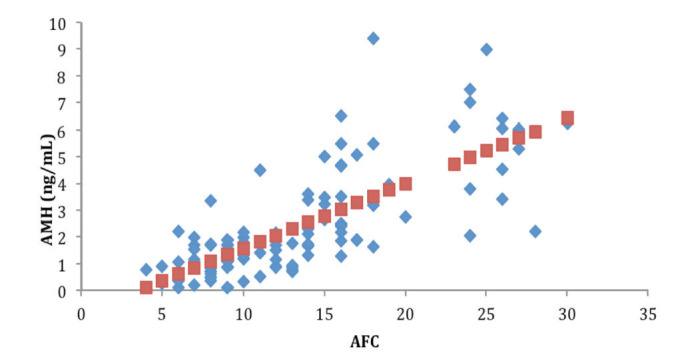
Correlation between Anti-Mullerian (AMH) and Antral Follicle Count (AFC).

**Figure 3:**
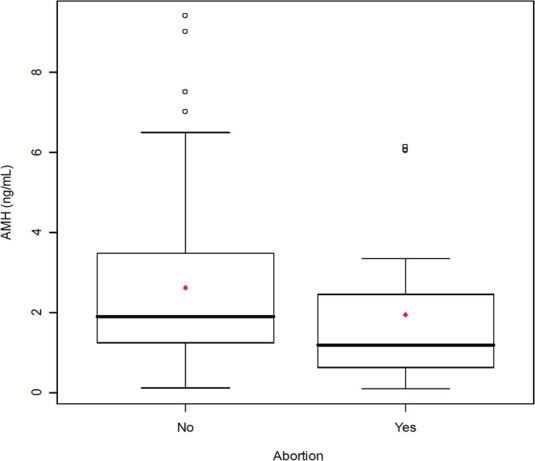
Serum anti-Mullerian hormone (AMH) comparison between infertile patients with abortion and infertile patients without abortion. The box represents the interquartile range that contains the 50% of values. The whiskers are line that extend from the box to highest and lowest values, exclude outliers. A line across the box indicates the median. *P*<.05, Student’s t-test.

**Figure 4:**
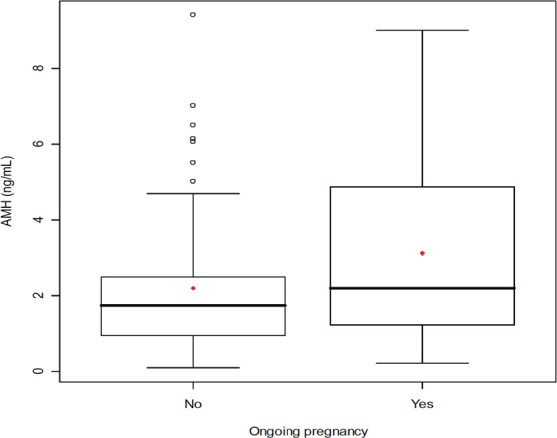
Serum anti-Mullerian hormone (AMH) comparison between infertile patients with ongoing pregnancy and infertile patients no ongoing pregnancy. The box represents the interquartile range that contains the 50% of values. The whiskers are line that extend from the box to highest and lowest values, exclude outliers. A line across the box indicates the median. *P*<.05, Student’s t-test.

When the 112 patients evaluated was observed a significant relationship of AMH with age (r =-0.37 *P* <.01) (Fig. 1A), with FSH (r =-0.22, *P* <.01) (Fig. 1B), with AFC (r = 0.74, *P* <.00001) (Fig. 2), with smoking (*P* <.009), with SOP (*P* <.00001), with the total dose of the drug during stimulation ovarian (r =-0.36, *P* <.0004), with abortion (*P* <.05) (Fig. 3) and with the ongoing pregnancy (*P* <.05) (Fig. 4).

## DISCUSSION

This study demonstrates that serum AMH level is an independent predictor of ongoing pregnancy in infertile women. Ovarian reserve is currently defined as an interplay between the quantity and quality of the follicles left in the ovary and several proxy variables for pool size are well described in the literature.

AMH can probably predict pregnancy, which is often used as a proxy for oocyte quality, is still a matter of debate (Ficicioglu *et al*., 2006; Lekamge *et al*., 2007; Broer *et al.*, 2009).

An explanation for our finding that AMH level can predict ongoing pregnant in this cohort could be the fact that higher AMH values are associated with a higher oocyte yield in IVF treatment.

This higher oocyte yield consequently results in higher chances of pregnancy (Broer *et al.*, 2011; Fleming *et al.*, 2006). Similarly, another possible explanation of our result is the positive correlation between the serum AMH level and embryo score quality (Silberstein *et al.*, 2006) although another study (Lekamge *et al.*, 2007) no correlation was found. It is also observed a correlation between miscarriages with serum levels of AMH (Elter *et al.*, 2005) that can be explained by increased rate of fetal aneuploidy indirectly related to the embryo score.

This study does not itself explain the physiological basis of these findings. We postulate, however, that continued recruitment of additional antral follicles during the stimulatory phase of IVF results in higher AMH levels in individuals destined to produce better quality embryos and to have a better reproductive outcome.

Comparisons between the published data are therefore difficult to make due to their heterogeneity. More research will be required to confirm this finding and to explore its etiology.

In our study, total consumption of the gonadotropin dose was statistically correlated with AMH. The relationship between serum AMH levels and controlled ovarian stimulation outcome that we have observed is in agreement with previous studies on serum AMH levels. Serum AMH seems to reflect the follicular pool, and its production is independent of the gonadotropin-dependent indicators of ovarian reserve. Another correlation is demonstrated in this study of AMH with polycystic ovarian syndrome (PCOS). The women with PCOS have elevated concentrations of AMH due probably an abnormal activity of the GCs, circulating androgen levels and the follicle excess seen on ultrasonographic examination (Laven *et al.*, 2004; Pigny *et al.*, 2006; Pellatt *et al.*, 2007; Das *et al.*, 2008; Li *et al.*,2011).

The negative impact of both male and female smoking on spontaneous and assisted conception rates has been largely demonstrated (Dechanet *et al.*, 2011; Waylen *et al.*, 2009). Tobacco consumption can result in an alteration of ovarian reserve, negative influence on the uterine receptiveness (Soares *et al.*, 2007) and possibly on the fertilization and embryo development (Hassa *et al.*, 2007; Jennings *et al.*, 2011; Huang *et al.*, 2009). Concerning IVF cycles, at present, the majority of the studies available report that smokers experience lower ovarian response to COH (Neal *et al.*, 2005; Weigert *et al.*, 1999), and finally lower implantation rate (Dessolle *et al.*, 2011).

## CONCLUSION

AMH is a marker of quantitative and qualitative aspects of the ovarian reserve.

In summary, AMH is now our main method of determining ovarian reserve and selecting our pretreatment counseling and choice of infertility treatment. We believe it to be the most informative serum marker available and that it should be considered an important part of any contemporary reproductive medicine practice.
